# Suicide by Clinic-Referred Transgender Adolescents in the United Kingdom

**DOI:** 10.1007/s10508-022-02287-7

**Published:** 2022-01-18

**Authors:** Michael Biggs

**Affiliations:** Department of Sociology and St Cross College, University of Oxford, 42 Park End Street, Oxford, OX1 1JD UK

## Introduction

Surveys show that adolescents who identify as transgender are vulnerable to suicidal thoughts and self-harming behaviors (dickey & Budge, 2020; Hatchel et al., 2021; Mann et al., 2019). Little is known about death by suicide. This Letter presents data from the Gender Identity Development Service (GIDS), the publicly funded clinic for children and adolescents aged under 18 from England, Wales, and Northern Ireland. From 2010 to 2020, four patients were known or suspected to have died by suicide, out of about 15,000 patients (including those on the waiting list). To calculate the annual suicide rate, the total number of years spent by patients under the clinic’s care is estimated at about 30,000. This yields an annual suicide rate of 13 per 100,000 (95% confidence interval: 4–34). Compared to the United Kingdom population of similar age and sexual composition, the suicide rate for patients at the GIDS was 5.5 times higher. The proportion of patients dying by suicide was far lower than in the only pediatric gender clinic which has published data, in Belgium (Van Cauwenberg et al., 2021).

### Suicidality in Transgender Adolescents

“About half of young trans people…attempt suicide,” declared the United Kingdom Parliament’s Women and Equalities Committee (2015). Similar figures are cited by news media and campaigning organizations. The *Guardian* reported Stonewall’s statistic that “almost half” of transgender young people “have attempted to kill themselves” (Weale, 2017). “Fifty percent of transgender youth attempt suicide before they are at age 21” stated the mother of the most famous transgender youth in the English-speaking world (Jennings & Jennings, 2016). As a transgender theologian has observed, “the statistic about suicide attempts has, in essence, developed a life of its own” (Tanis, 2016).

Representative surveys of students in high schools provide one source of evidence for this statistic. In New Zealand, 20% of transgender students reported attempting suicide in the past 12 months, compared to 4% of all students (Clark et al., 2014). In the United States, 15% of transgender students reported a suicide attempt requiring medical treatment in the last 12 months, compared to 3% of all students (Centers for Disease Control & Prevention, 2018; Jackman et al., 2021; Johns et al., 2019). In another American survey, 41% of transgender students reported having attempted suicide during their lifetime, compared to 14% of all students (Toomey et al., 2018).

To what extent are self-reported suicide attempts reflected in fatalities? The connection is not straightforward. Respondents who report suicide attempts are not necessarily indicating an intent to die. One survey of the American population found that almost half the respondents who reported attempting suicide subsequently stated that their action was a cry for help and not intended to be fatal (Nock & Kessler, 2006). In two small samples of non-heterosexual youth, half the respondents who initially reported attempting suicide subsequently clarified that they went no further than imagining or planning it; for the remainder who did actually attempt suicide, their actions were usually not life-threatening. To an extent, then, “the reports were attempts to communicate the hardships of lives or to identify with a gay community” (Savin-Williams, 2001). Although such elaborate survey methods have not been used to study transgender populations, there is anecdotal evidence for a similar disjuncture. The pediatric endocrinologist who established the first clinic for transgender children in the United States stated that “the majority of self-harmful actions that I see in my clinic are not real suicide attempts and are not usually life threatening” (Spack, 2009).

Suicide mortality has been studied in the transgender population using registry data. The annual suicide rate is calculated by dividing the number of suicides by the total number of years each person was at risk. An individual who was observed for 20 years, for instance, contributes 20 person-years to the denominator. The largest study covers over 8,000 patients who visited the gender clinic in Amsterdam from 1972 to 2017 (Wiepjes et al., 2020). The annual suicide rate was 29 per 100,000 for transmen, quadruple the rate for the female population, and 64 for transwomen, quadruple the rate for the male population. A Swedish study of 324 individuals who had undergone genital surgery between 1973 and 2003 found much higher annual suicide rates: 250 per 100,000 for transmen, 43 times the rate for matched female controls, and 285 for transwomen, 16 times the rate for matched male controls (M. Boman, personal communication, 12 April 2021; Dhejne et al., 2011). Only one published study has reported suicide fatalities among transgender adolescents. Belgium’s pediatric gender clinic provided counseling to 177 youth aged from 12 to 18 years, who had been referred between 2007 and 2016: five of them (2.8%) committed suicide (Van Cauwenberg et al., 2021). The mean age of referral was 15, implying a mean duration of 3 years before transition to an adult clinic, which translates to an annual suicide rate of 942 per 100,000. This is the highest suicide mortality recorded for any transgender population.

## Method

This Letter estimates the suicide rate at the world’s largest pediatric gender clinic. Based in London, the GIDS is part of the Tavistock and Portman NHS Foundation Trust, and serves youth under 18 from England, Wales, and Northern Ireland who are “experiencing difficulties with their gender identity development” (Carmichael & Davidson, 2009). Like all such services throughout Western Europe and North America, it has experienced enormous growth; referrals increased from 100 in 2009 to a peak of 2700 in 2019. The waiting list in April 2021 exceeded 5300.

The GIDS patients manifest typically high rates of self-harming behavior. In a sample of 900 adolescents (aged from 13 to 17) admitted to the clinic from 2009 to 2017 and given the Youth Self-Report questionnaire, 44% answered that they sometimes or very often “deliberately try to hurt or kill myself” (de Graaf et al., 2020). Unfortunately, both behaviors are combined in this question. In a different sample of over 700 children and adolescents (aged from 4 to 17) assessed by the GIDS in 2012 and 2015, 10% were flagged by clinicians as having attempted suicide (Morandini et al., 2021).

### Suicides

Since the early 2000s, the National Health Service has implemented mandatory reporting of “serious incidents” (Department of Health, 2001, 2010). The death of any patient—including those on the waiting list—suspected to be suicide is reported to the Tavistock’s Board of Directors. The Tavistock cooperates with a comprehensive surveillance system for every death classified as suicide or (after an open verdict by the coroner) probable suicide in the United Kingdom (National Confidential Inquiry into Suicide & Homicide by People with Mental Illness, 1999; National Confidential Inquiry into Suicide and Safety in Mental Health, 2019). Papers for the Tavistock’s Board meetings are available from April 2007 onwards; those not on the Trust’s website were acquired by a Freedom of Information request. The pdf files of the *Agenda and Papers* (through September 2021) were searched for the keyword “suicid”; all 442 instances were inspected. From 2007 to 2020, four patients of the GIDS died by suspected suicide: two on the waiting list, in 2016 and 2017; and two after having been seen, in 2017 and 2020. The last case was described as “likely” to be suicide, because the inquest has not yet been held. These figures were confirmed by Freedom of Information requests to the Tavistock.

Triangulation is possible from two sources. Comprehensive mortality data on all adolescents aged from 10 to 19 who committed suicide in the United Kingdom from 2014 to 2016 include five transgender individuals (Rodway et al., 2020). Due to confidentiality restrictions, it is not possible to disaggregate these further by age or by country. Presumably, one of these is the patient of GIDS who died in 2016. The remaining four might have been 18 or 19—the risk of suicide increases significantly in the late teens—or might have lived in Scotland. Alternatively, they might have been eligible for the GIDS but had not sought a clinical referral (made by the local Child and Adolescent Mental Health Service, the child’s general practitioner, social worker, or teacher) or had not obtained it.

Another source is the Transgender Day of Remembrance website, which aims to record all deaths by suicide or violence (Metcalfe, 2021). For the United Kingdom between 2007 and 2020, the website names 3 adolescents under the age of 18 who committed suicide. One was one of the GIDS patients (the match is certain because they were named in the *Agenda and Papers*). The other two had no involvement with the GIDS (or any other gender clinician), as was evident from their inquests, though one was under the psychiatric care of another NHS Trust (BBC News, 2021; Bunyan, 2008). In addition, the website lists suicides by two “young” transgender people, sourced from Twitter, without information on their name or age. In one case, it is not clear whether the person lived in the United Kingdom.

### Patients

With suicides as numerator, two denominators are relevant. Because comprehensive data on patient numbers became available from 2010, the period will be the 11 years from 2010 to 2020. (These are financial years; thus, 2020 runs from April 2020 to March 2021.) The first denominator is the total number of individual patients, estimated by summing the annual number of referrals to the GIDS from 2010 to 2020—excluding those aged 18 or over, as they are not accepted. The total number is 15,032. This sum omits patients at the clinic who had been referred before 2010, and so is a slight underestimate. (The Online Supplement provides full details.)

The second denominator is the total number of patient-years: the sum of the number of years spent by each individual as a patient of the GIDS. The number of patients seen by the GIDS each year was available from 2014 to 2020. Before 2014 only the number of patients first seen was available. From 2014 to 2016, the number of patients seen was consistently double the number first seen, and so the former number for 2010 to 2013 was estimated by doubling the latter. All these numbers exclude patients on the waiting list. The number waiting at the beginning of each year from 2016 to 2020 was obtained by Freedom of Information request. Before then the number was not available, and so must be treated as zero. This leads to an underestimate, of course, but the waiting list became appreciable only from 2015. The total number of patient-years over this period is estimated as 30,080. In other words, patients spent on average 2 years at the GIDS (including time on the waiting list). Time on the waiting list contributed 41% of the total patient-years.

## Results

From 2010 to 2020, the four suicide deaths equate to 0.03% of the 15,032 patients. Taking the denominator as 30,080 patient-years, the annual suicide rate is calculated as 13 per 100,000 (95% confidence interval: 4 to 34 per 100,000). For comparison, the annual suicide rate in England and Wales between 2010 and 2020 for adolescents aged from 15 to 19 years averaged 4.7 (Office for National Statistics, 2021). This does not quite correspond to the age range of the GIDS patients, however. At referral, the patients ranged in age from 3 to 17 years; only 7% were younger than 10. The mean was 14 years and the median 15. Most patients stay with the GIDS until transitioning to an adult service. Therefore, the average age of patients at any point in time will lie somewhere between 14 and 17. A better comparison is therefore the overall suicide rate for adolescents aged from 14 to 17 (available only for the entire United Kingdom for 2015–2017), which was 2.7 per 100,000 (Office for National Statistics, 2018; Rodway et al., 2020). Comparison should also account for the difference between the sexes, because males are more likely to commit suicide than females. Of the GIDS patients, 69% were female. Adjusting for sex, the GIDS patients were 5.5 times more likely to commit suicide than the overall population of adolescents aged 14 to 17.

## Discussion

How reliable are these estimates? The chief uncertainty about the numerator is whether the fourth death will be ruled as suicide when the inquest is eventually held. It could be speculated that there were further suicides unknown to the Tavistock and to the National Confidential Inquiry into Suicide and Safety in Mental Health. All that can be said is that the single suicide by a GIDS patient from 2014 to 2016 is not out of line with comprehensive mortality data on suicides by transgender adolescents in the United Kingdom which counted five suicides in a longer age range and wider geographical area. The denominator for the annual suicide rate, however, is pieced together from various series and so is inevitably approximate. Statistics from the early 2010s are less reliable, though they make only a small contribution to the grand total; the last three years contribute more than half of the total number of patient-years. The most significant limitation is the lack of information on the age and sex of all the patients who committed suicide.

Direct comparison can be made with the Belgian pediatric gender clinic (Van Cauwenberg et al., 2021). Its annual suicide rate was about 70 times greater than the rate at the GIDS. This is especially puzzling because patients at the Belgian clinic scored better, on average, than those at the GIDS on tests of psychological functioning (de Graaf et al., 2018). The explanation for the huge disparity in suicide is not clear. The Amsterdam’s clinic annual suicide rate was four times greater than the rate at the GIDS. The higher rate is not surprising, however, because the Dutch clinical population was dominated by older adults: the median age at first visit was 25 (Wiepjes et al., 2020). Suicide rates peak in middle age, and so a population of older adults would be at higher risk than a population of adolescents.

The suicide rate of the GIDS patients is not necessarily indicative of the rate among all adolescents who identify as transgender. On the one hand, individuals with more serious problems (and their families) would be particularly motivated to seek referral and more likely to obtain it, and so the clinical subset would be more prone to suicide. One study suggests that a child who frequently attempted suicide was more readily referred to the GIDS (Carlile et al., 2021). On the other hand, young people facing hostility from their families would be less able to seek referral, and this hostility could make them especially vulnerable to suicide.

Taking into account these limitations, the estimated suicide rate at the GIDS provides the strongest evidence yet published that transgender adolescents are more likely to commit suicide than the overall adolescent population. The higher risk could have various causes: gender dysphoria, accompanying psychological conditions, and ensuing social disadvantages such as bullying. Studies of young people referred to the GIDS in 2012 and 2015 found a high prevalence of eating disorders, depression, and autism spectrum conditions (ASC) (Holt et al., 2016; Morandini et al., 2021)—all known to increase the probability of suicide (Simon & VonKorff, 1998; Smith et al., 2018). Eating disorders and depression could be consequences of transgender identity and its ensuing social repercussions, but this is implausible for ASC insofar as it originates in genes or the prenatal environment. From a sample of over 700 referrals to the GIDS in 2012 and 2015, 14–15% were diagnosed with ASC (Morandini et al., 2021). This compared to 0.8–1.1% of students in England (Department for Education, 2012, 2015). The association between autism and gender dysphoria is found in many populations (Socialstyrelsen, 2020; Warrier et al., 2020). Autism is known to increase the risk of suicide mortality, especially in females (Hirvikoski et al., 2016; Kirby et al., 2019; Socialstyrelsen, 2020). To some extent, therefore, the elevated suicide rate for transgender youth compared to their peers reflects the higher incidence of ASC. The same holds for other psychiatric disorders associated with gender dysphoria (Dhejne et al., 2016). Ideally, the suicide rate for patients of the GIDS would be compared to the suicide rate for patients in contact with other NHS mental health services, but the latter rate is not available.

One final caveat is that these data shed no light on the question of whether counseling or endocrinological interventions—gonadotropin-releasing hormone agonist or cross-sex hormones—affect the risk of suicide (Biggs, 2020; Turban et al., 2020). Although two out of the four suicides were of patients on the waiting list, and thus would not have obtained treatment, this is not disproportionate: the waiting list contributed nearly half of the total patient-years.

### Conclusion

Data from the world’s largest clinic for transgender youth over 11 years yield an estimated annual suicide rate of 13 per 100,000. This rate was 5.5 times greater than the overall suicide rate of adolescents of similar age, adjusting for sex composition. The estimate demonstrates the elevated risk of suicide among adolescents who identify as transgender, albeit without adjusting for accompanying psychological conditions such as autism. The proportion of individual patients who died by suicide was 0.03%, which is orders of magnitude smaller than the proportion of transgender adolescents who report attempting suicide when surveyed. The fact that deaths were so rare should provide some reassurance to transgender youth and their families, though of course this does not detract from the distress caused by self-harming behaviors that are non-fatal. It is irresponsible to exaggerate the prevalence of suicide. Aside from anything else, this trope might exacerbate the vulnerability of transgender adolescents. As the former lead psychologist at the Tavistock has warned, “when inaccurate data and alarmist opinion are conveyed very authoritatively to families we have to wonder what the impact would be on children’s understanding of the kind of person they are…and their likely fate” (Wren, 2015).

## Supplementary Information

Below is the link to the electronic supplementary material.

Supplementary file1 (PDF 105 kb)
