# Supplementary material for: Suicide by Clinic-Referred Transgender Adolescents in the United Kingdom
Source: Arch Sex Behav. 2022 Jan 18;51(2):685–90. doi: 10.1007/s10508-022-02287-7 (PMC8888486; doi:10.1007/s10508-022-02287-7)
Supplement: Supplementary file 1 — Supplementary file1 (PDF 105 kb) [file 10508_2022_2287_MOESM1_ESM.pdf]

Suicide by Clinic-Referred Transgender Adolescents in the United Kingdom
Michael Biggs
Archives of Sexual Behavior, 2022

ONLINE SUPPLEMENT

1. Suicides

Patients known or suspected to have died by suicide are reported to the Tavistock and Portman NHS Foundation Trust's Board of Directors. I searched pdf files of the *Agenda and Papers* from April 2007 to September 2021 for every instance of the keyword "suicid" and found four suicides by patients of the Gender Identity Development Service (GIDS).

May 2016: on waiting list (*Agenda and Papers*, May 2017, p. [21])
31 May 2017: being seen (*Agenda and Papers*, November 2017, p. 214)
November 2017: on waiting list (*Agenda and Papers*, March 2018, p. 137)
October–December 2020: being seen (*Agenda and Papers*, March 2021, p. 90)

These cases were confirmed by Freedom of Information requests (FOI 18-19180, 19-20375, 21-22068).

There is evidence for one earlier suicide, as in 2016 the GIDS website stated:

> "suicide is extremely rare, with one case in the service in the last decade, of a young person in an inpatient ward who was referred with severe psychiatric difficulties." (https://web.archive.org/web/20161125121442/https://gids.nhs.uk/evidence-base).

When this statement was written is unknown, though the preceding sentence cites an article published in 2015. The description of this suicide does not appear to match the suicide that occurred in May 2016, and so presumably it occurred before 2007.

2. Total number of patients

The annual number of referrals were reported on the website of the GIDS. I subtract the number aged 18 or over, as those referrals are rejected.

2010/11: 139
2011/12: 204
2012/13: 307
2013/14: 463
2014/15: 673
2015/16: 1,373
2016/17: 1,986
(https://web.archive.org/web/20170613221701/https://gids.nhs.uk/number-referrals)
2017/18: 2,356
(https://web.archive.org/web/20180527201924/https://gids.nhs.uk/number-referrals)
2018/19: 2,560
(https://web.archive.org/web/20190722103107/https://gids.nhs.uk/number-referrals)
2019/20: 2,668
(https://web.archive.org/web/20200701000226/https://gids.nhs.uk/number-referrals)

2020/21: 2,303
(https://web.archive.org/web/20210630151630/https://gids.nhs.uk/number-referrals)

The total of 15,032 is a slight underestimate, because it omits those patients who had been referred before 2010/11 but who were still at the GIDS in this period. Note that about 1.5% of the total patients came from the Republic of Ireland.

3. Annual number of patients seen
The Tavistock Trust reported the annual number of patients seen by the GIDS from 2014 onwards:

2014/15: 1,027
2015/16: 1,449
2016/17: 2,292
2017/18: 2,685
2018/19: 2,717
(Board of Directors, *Agenda and Papers*, May 2019, p. 37)
2019/20: 2,828
(Freedom of Information request FOI 20-21258)
2020/21: 2,846
(Freedom of Information request FOI 21-22153)

Before 2014, figures are available only for the number of patients *first* seen:

2010/11: 164
2011/12: 181
2012/13: 244
2013/14: 356
2014/15: 522 (51% of patients seen)
2015/16: 745 (51% of patients seen)
2016/17: 1,223 (53% of patients seen)
2017/18: 1,011 (38% of patients seen)
(Freedom of Information request FOI 18-19206)

I estimate the number of patients seen from 2011/12 to 2013/14 as double the number first seen, following the ratio in the following three years.

4. Annual number of patients on the waiting list
The number of patients on the waiting list for the GIDS at the beginning of the financial year was obtained from a Freedom of Information request:

April 2016 621
April 2017 1,255
April 2018 2,350
April 2019 3,487
April 2020 4,633
(Freedom of Information request FOI 20-21258)

Numbers before 2016 are not available. I treat them as zero, which will lead to an underestimate, though the waiting list became appreciable only in 2015.
